# Redox-Mediated Mechanisms Fuel Monocyte Responses to CXCL12/HMGB1 in Active Rheumatoid Arthritis

**DOI:** 10.3389/fimmu.2018.02118

**Published:** 2018-09-19

**Authors:** Valentina Cecchinato, Gianluca D'Agostino, Lorenzo Raeli, Alessandra Nerviani, Milena Schiraldi, Gabriela Danelon, Antonio Manzo, Marcus Thelen, Adrian Ciurea, Marco E. Bianchi, Anna Rubartelli, Costantino Pitzalis, Mariagrazia Uguccioni

**Affiliations:** ^1^Faculty of Biomedical Sciences, Institute for Research in Biomedicine, Università della Svizzera italiana, Bellinzona, Switzerland; ^2^Barts and The London School of Medicine and Dentistry, William Harvey Research Institute, Queen Mary University of London, London, United Kingdom; ^3^Division of Rheumatology, Rheumatology and Translational Immunology Research Laboratories (LaRIT), IRCCS Policlinico San Matteo Foundation and University of Pavia, Pavia, Italy; ^4^Department of Rheumatology, University Hospital Zurich, Zurich, Switzerland; ^5^San Raffaele University and Scientific Institute, Milan, Italy; ^6^Cell Biology Unit, IRCCS Ospedale Policlinico San Martino, Genoa, Italy; ^7^Department of Biomedical Sciences, Humanitas University, Milan, Italy

**Keywords:** cell migration, monocytes, CXCL12, HMGB1, thioredoxin, rheumatoid arthritis

## Abstract

Chemokine synergy-inducing molecules are emerging as regulating factors in cell migration. The alarmin HMGB1, in its reduced form, can complex with CXCL12 enhancing its activity on monocytes via the chemokine receptor CXCR4, while the form containing a disulfide bond, by binding to TLR2 or TLR4, initiates a cascade of events leading to production of cytokines and chemokines. So far, the possibility that the CXCL12/HMGB1 heterocomplex could be maintained in chronic inflammation was debated, due to the release of reactive oxygen species. Therefore, we have assessed if the heterocomplex could remain active in Rheumatoid Arthritis (RA) and its relevance in the disease assessment. Monocytes from RA patients with active disease require a low concentration of HMGB1 to enhance CXCL12-induced migration, in comparison to monocytes from patients in clinical remission or healthy donors. The activity of the heterocomplex depends on disease activity, on the COX2 and JAK/STAT pathways, and is determined by the redox potential of the microenvironment. In RA, the presence of an active thioredoxin system correlates with the enhanced cell migration, and with the presence of the heterocomplex in the synovial fluid. The present study highlights how, in an unbalanced microenvironment, the activity of the thioredoxin system plays a crucial role in sustaining inflammation. Prostaglandin E2 stimulation of monocytes from healthy donors is sufficient to recapitulate the response observed in patients with active RA. The activation of mechanisms counteracting the oxidative stress in the extracellular compartment preserves HMGB1 in its reduced form, and contributes to fuel the influx of inflammatory cells. Targeting the heterocomplex formation and its activity could thus be an additional tool for dampening the inflammation sustained by cell recruitment, for those patients with chronic inflammatory conditions who poorly respond to current therapies.

## Introduction

The concomitant presence of pro-inflammatory cytokines (1, 2), growth factors (3), prostaglandins (4), as well as chemokines (5) orchestrate the characteristic features of Rheumatoid Arthritis (RA). Several inflammatory chemokines, key regulators of leukocyte migration (6), are present at high levels in RA synovial fluid and tissue, with some of them accounting for the recruitment of monocytes/macrophages (7), or indicating disease activity (8). The analysis of the synovial cell infiltrate shows that the number of monocytes/macrophages increases in joints with active inflammation. Conversely, the reduction in the amount of infiltrating monocytes is associated with successful therapy, making this cell type a good biomarker of RA disease activity (9). In particular, monocyte migration into human synovium is driven by CXCL12 (10), while the organization of lymphoid-like structures is controlled by CXCL13 and CCL21 (11). An impressive amount of preclinical and clinical evidence has progressively validated the role of chemokines and their receptors in immune-mediated diseases (12), which led to the development of several chemokine receptor blocking agents, initially tested with success in RA pre-clinical models (13). However, most of these competitive chemokine receptor antagonists have disappointed when their efficacy was assessed in clinical trials (5, 14).

The influx of monocytes into the inflammatory environment favors the release of damage-associated molecular pattern molecules (DAMPs), which are endogenous danger signals released by necrotic cells or by immune cells stimulated by pro-inflammatory mediators (15). High Mobility Group Box 1 (HMGB1) originally identified as chromatin-associated protein (16), is a DAMP released by necrotic cells after tissue injury (17), or secreted by activated inflammatory cells, and can be present in the microenvironment in multiple redox forms. Whether and how the redox status of HMGB1 is modulated by extracellular catalysts was so far unknown, and have been elucidated in the present study. The form containing a disulfide bond, between cysteine 23 and 45, by binding to TLR2, or TLR4, initiates a cascade of events leading to NF-κB translocation to the nucleus and transcription of cytokine and chemokine genes (18, 19). On the contrary, reduced HMGB1 modulates autophagy through the interaction with the receptor for advanced glycation end products (RAGE) (20).

Our group has described that, when fully reduced, HMGB1 can also form a complex with CXCL12, which is much more powerful than CXCL12 alone in inducing the migration of human monocytes via its selective receptor CXCR4. A suboptimal concentration of CXCL12 that *per se* would not induce monocyte migration, when combined with HMGB1 is able to recruit monocytes *in vitro* and *in vivo* (21, 22). Recent data demonstrate that the CXCL12/HMGB1 heterocomplex, via CXCR4, can also coordinate tissue regeneration (23, 24).

CXCR4 is a G protein-coupled receptor (6) that initiates a series of signaling events. The canonical signaling pathway leads to chemotaxis and expression of specific genes (25). In addition, CXCL12 binding to CXCR4 (26) activates the JAK-STAT pathway, which contributes to the induction of cell migration (27). The involvement of JAK in the signaling triggered by several cytokines, interferons, and growth factors has driven the development of several inhibitors for the treatment of inflammatory disorders, including RA (28).

Among the principal mediators of inflammation in RA, Prostaglandin E2 (PGE_2_) represents the target of non-steroidal anti-inflammatory drugs and selective cyclooxygenase-2 (COX2) inhibitors, such as celecoxib (29). The binding of PGE_2_ to its receptors leads to the activation of the PI3K/AKT signaling pathways, as well as to the production of cyclic AMP, promoting a cascade of events that contributes to the unbalanced cellular and molecular microenvironment (30), which occurs in RA.

The redox proteins Thioredoxin-1 (Trx) and Thioredoxin reductase (TrxR) provide resistance against oxidative stress and have been recently related to RA severity (31, 32). Of note, Trx and TrxR are secreted by a variety of normal and neoplastic cells (33, 34) and catalytically interact with proteins of the extracellular face of plasmamembrane, including cytokine receptors, changing their redox status and consequently their functions (35).

Although CXCL12, HMGB1, JAK, COX2, and PGE_2_ have all been associated with RA, their exact mechanistic connections are unclear. This has clinical relevance, as current treatments in RA can achieve substantial benefit for the patient, and several biomarkers can define clinical remission (36), but the final goal of blocking inflammation is not achieved in a considerable number of patients. This suggests that the different ligands and receptors might be redundant, interconnected, or modulated by undisclosed mechanisms.

The present study describes how in patients with active RA the redox state of the environment, and the cell-signaling cascade support the activity of the CXCL12/HMGB1 heterocomplex on monocytes, sustaining the inflammatory condition. These findings contribute to further understand RA pathology, how the redox form of HMGB1 is controlled in the inflammatory environment, and indicate novel therapeutic interventions for those patients not responding to conventional treatments.

## Materials and methods

### Patients

A total of 41 RA patients undergoing routine disease assessment at the Barts Health NHS Trust (London, UK) and at the University Hospital (Zurich, CH) were enrolled in the study. All patients fulfilled the American College of Rheumatology 1987 revised criteria for the classification of RA (37) and were at different stages of the disease. Newly diagnosed patients were treatment-naïve, while patients with long-standing RA received standard treatment with conventional synthetic or biologic disease modifying anti-rheumatic drugs (DMARDs) as per local guidelines. Disease activity was assessed by using the 28-joint Disease Activity Score (DAS28) on the day of peripheral blood or synovial fluid collection. Patients were divided into two groups accordingly to the DAS28 cutoffs indicated by the recommendations of the American College of Rheumatology (38): DAS28 >3.2 for patients with active disease (moderate to high disease activity), and DAS28<2.6 for patients in clinical remission. We collected monocytes from 15 patients, synovial fluid from 26 patients, and plasma from 10 patients. The study was approved by the Ethical Committees of the King's College Hospital (Rec. No. 05/Q0703/198), the East London & The City Research Authority (Rec No. 07/Q0605/29), and of the Canton Zurich (No. EK-515). Written informed consent was obtained from every patient, and all samples were rendered anonymous. Demographic, clinical and serological features of the patients included in the study are reported in Table 1.

**Table 1:** Demographic, clinical, and serological features of the RA patients included in the study.

**Demographics**	**Early RA treatment-naïve (*n* = 26 synovial fluids)**	**DAS28<2.6 (*n* = 6)**	**DAS28>3.2 (*n* = 9)**
Female % *(n)*	80.8% (21)	66.7% (4)	66.7% (6)
Age years *(mean ± SD)*	58.5 ± 14.7	51 ± 13.4	63.3 ± 13.0
**BIOCHEMICAL AND CLINICAL PARAMETERS**			
ESR mm/h *(mean ± SD)*	46.6 ± 32.1	15 ± 9.4	44.4 ± 48.4
CRP mg/l *(mean ± SD)*	29.1 ± 36.2	0.0 ± 0.0	51.3 ± 106.7
RF + % *(n)*^a^	70.8% (17)	66.7% (4)	55.6% (5)
Anti-CCP + % *(n)*^a^	70.8% (17)	*N.D*.	*N.D*.
TJ/28 joints *(mean ± SD)*	10.9 ± 7.5	0 ± 0	14.1 ± 8.2
SJ/28 joints *(mean ± SD)*	7.8 ± 7.2	0.4 ± 0.9	15.6 ± 9.1
VAS GH patient *(mean ± SD)*	69.1 ± 26.8	*N.D*.	*N.D*.
DAS28 *(mean ± SD)*	5.88 ± 1.39	2.2 ± 0.3	5.3 ± 1.3
DAS28 *(range)*	1.88 - 8.41	1.9-2.56	3.3-7

*RA, Rheumatoid Arthritis; SD, Standard Deviation; ESR, Erythrocyte Sedimentation Rate; CRP, C-Reactive Protein; RF, Rheumatoid Factor; CCP, Cyclic Citrullinated Peptide; TJ, Tender Joints; SJ, Swollen Joints; VAS, Visual Analog Score; GH, Global Health; DAS, Disease Activity Score; N.D., not determined*.

a *RF and CCP titres available for 24/26 patients*.

### Blood, synovial fluid collection, and cell isolation

Sixteen milliliters of whole blood from RA patients was collected in BD Vacutainer® CPT™ Cell preparation tubes (362782, BD Biosciences, San Jose, CA). Blood from healthy donors (HD) was provided as buffy-coats by the Central Laboratory of Swiss Red Cross (Basel, Switzerland). All samples were processed within 24 h after blood withdrawal. Peripheral blood mononuclear cells (PBMCs) were isolated using Ficoll-hypaque density centrifugation. Monocytes, CD14+, were isolated by positive immunoselection procedure (130-050-201, CD14 MicroBeads, Miltenyi Biotec, Germany) according to the manufacturer's instructions. CD14+ monocytes from HD were cultured for 6 h at a density of 1.7 x 10^6^ cell/ml in RPMI-1640 (31870) supplemented with 10% Fetal Bovine Serum (16000-044), 1x non-essential amino acids (11140-035), 1 mM Sodium pyruvate (11360-039), 20 mM GlutaMAX (35050-038), 50 μM β-Mercaptoethanol (31350-010), Penicillin 50 U/ml – Streptomycin 50 μg/ml (15070-063), all from Gibco ThermoFisher Scientific, Switzerland, in the absence or presence of PGE_2_, at the final concentration of 1 nM, or IL-6 and IL-1β at the final concentration of 20 ng/ml. For the experiments using HD monocytes, COX2, or JAK2 inhibitors were added to the cells together with PGE_2_ during the 6 h culture. Synovial fluid samples were obtained by aspiration of joint fluids from inflamed knees and stored at −80°C until usage.

### Reagents

CXCL12 was synthesized using tBoc solid-phase chemistry (39). Full-length HMGB1 was produced and stored in buffer containing DTT (40) at the Institute of Research in Biomedicine protein facility, or purchased (1690-HMB, R&D Systems, Minneapolis, MN). PGE_2_ (14010) was purchased from Cayman Chemical (Ann Arbor, MI). Recombinant human IL-6 (6206-IL) and recombinant human IL-1β (201-LB) were purchased (R&D Systems, Minneapolis, MN). TG101348 was from Selleck Chemicals (S2736, Houston, TX), celecoxib (PZ0008), and Auranofin (A6733) were purchased from Sigma-Aldrich (Saint Louis, MO). Mouse anti-human CXCR4 APC-conjugated (555976, BD Biosciences, San Jose, CA), mouse anti-human CXCL12 (MAB350, R&D Systems, Minneapolis, MN), rabbit anti-human HMGB1 (Ab18256, Abcam, Cambridge, UK), mouse anti-HMGB1 (HM-901, HMGBiotech, Milan, Italy), and rabbit anti-human thioredoxin (2429, Cell Signaling technology, Leiden, The Netherlands).

### Flow cytometry analysis

All antibodies were diluted 1:50, and monocytes stained with anti-CXCR4-APC antibody for 20 min at 4°C. Samples were acquired by using BD LSR Fortessa flow cytometer from BD Biosciences (San Jose, CA) and analyzed with the FlowJo software (FLOWJO LLC, Ashland, OR). The expression of CXCR4 was calculated as ratio between the Mean Fluorescence Intensity (MFI) detected in the stained samples on the unstained control samples performed for each condition.

### CXCR4 oligomerization determined by PLA

CXCR4 homodimerization was assessed using the Duolink® *in situ* Detection Reagent Red (DUO92008) using the anti-CXCR4 12G5 antibody diluted 1:100, and mouse PLUS (DUO092001) and MINUS (DUO092004) probes, all from Sigma-Aldrich (Saint Louis, MO). Briefly, monocytes from HD were seeded in eight well chambers (94.6150.801, Sarstedt, Nümbrecht, Germany), untreated or treated for 6 h with 1 nM PGE_2_ or 1 μM PGE_2_, fixed with 4% PFA in PBS for 10 min at 37°C. All subsequent steps were performed according to manufacturer instructions. Images were acquired with a Leica TCS SP5 confocal microscope (Leica Microsystems, Heerbrugg, Switzerland), using a 63x/1.4 N.A. objective, and analyzed using the open-source image analysis software Fiji (41).

### Chemotaxis assay

Migration of monocyte from RA patients or HD was analyzed using 48-well Boyden chambers (Neuro Probe, Cabin John, MD) with 5 μm pore size polyvinylpyrrolidone-free polycarbonate membranes as previously described (42). Briefly, 5 x 10^4^ monocytes were diluted in RPMI-1640 supplemented with 20 mM Hepes, pH7.4, and 1% pasteurized plasma protein solution (5% PPL SRK; Swiss Red Cross Laboratory, Berne, Switzerland). Cells were then added to the upper wells. After 90 min of incubation, the membrane was removed, washed on the upper side with phosphate-buffered saline (PBS), fixed, and stained. All assays were done in triplicate, and for each well the migrated cells were counted at 100-fold magnification in 5 randomly selected fields (5HPF). Spontaneous migration was determined in the absence of chemoattractant. The following reagents were added to the chemotaxis buffer (42) to assess their influence on monocyte migration: celecoxib at 1 nM and TG101348 at 30 nM.

### ELISA

Detection of Trx and TrxR reductase in RA plasma, synovial fluids, and monocyte supernatants was performed by ELISA following manufacturer instructions using the following kits: Trx (EK1254, Boster Biological Technology Co., Pleasanton, CA), and TrxR (ab192150, Abcam, Cambridge, UK). Detection of PGE_2_ in supernatants of monocytes was performed using Prostaglandin E2 EIA Kit-Monoclonal, following manufacturer instructions (CAY514010, Cayman chemical, Ann Arbor, MI). Monocytes from HD, untreated or treated with 1 nM PGE_2_ for 6 h were washed once with HBSS (14170120, Gibco ThermoFisher Scientific, Switzerland) to remove the exogenous given PGE_2_. In order to detect freshly synthetized PGE_2_, Trx, and TrxR, cells were supplemented with HBSS and cultured for additional 2 h, before collecting the supernatant.

### Hybrid ELISA

For the detection of the heterocomplex between CXCL12 and HMGB1, we performed a Hybrid ELISA as previously reported (43). Optical density was measured at 450 nm. All procedures were performed at room temperature.

### Western blot analysis

Monocytes from HD cultured for 6 h in the presence or absence of 1 nM PGE_2_, and freshly isolated monocytes from RA patients were suspended at the density of 1.7 x 10^6^ cell/ml in RPMI-1640 supplemented with 20 mM Hepes (pH 7.4), 1% pasteurized plasma protein solution (5% PPL SRK; Swiss Red Cross Laboratory, Berne, Switzerland), 1% protease inhibitor (P8340, Sigma-Aldrich, St. Louis, MO), and 300 nM HMGB1. Supernatants were collected after 30, 60, or 120 min. To preserve reduced residues, supernatants were alkylated with 20 mM iodoacetamide (C_2_H_4_INO) for 30 min at room temperature in the dark, and precipitated with ice-cold acetone. The dry pellets were loaded in non-reducing sample buffer for western blot analysis. The following reagents were added to the chemotaxis buffer to assess their influence on HMGB1 redox status: celecoxib at 1 nM, TG101348 at 30 nM, Auranofin at 2 μM.

The proteins were blotted onto a PVDF filter Immobilion-P (IPVH304F0, Millipore, Billerica, Massachusetts, MA), and HMGB1 content was detected using the anti-human HMGB1 from HMGBiotech, and by incubation with the secondary anti-mouse Goat horseradish peroxidase (HRP)-conjugate antibody (1706516, Bio-Rad, Hercules, CA) following manufacturer instructions. Super Signal (34077, Themo Scientific, Rockford, IL) was used as luminol-based enhanced chemiluminescent substrate for detecting HRP on the immunoblots. Reduced over oxidized form of HMGB1 was calculated on the densitometric values of the bands obtained.

### Immunohistochemistry

Formalin-fixed, paraffin-embedded synovial samples were obtained from RA patients fulfilling the American College of Rheumatology 1987 revised criteria (37) undergoing joint replacement, synovectomy, or arthroscopic biopsy. Tissue samples were cut to 5 μm slices and used for immunohistochemistry (IHC) (44). To detect HMGB1 and CXCL12, antigen retrieval was performed with Dako Target Retrieval Solution at pH6 (S1699, Dako, Agilent, Santa Clara, CA). To detect thioredoxin, antigen retrieval was performed with 10 mM Citric Acid, pH6. The concentrations of the primary antibodies used were as follows: mouse anti-human CXCL12 (MAB350, R&D Systems, Minneapolis, MN; 0.5 μg/ml), rabbit anti-human HMGB1 (Ab18256, Abcam, Cambridge, UK; 1 μg/ml), and rabbit anti-human thioredoxin (2429, Cell Signaling technology, Leiden, The Netherlands, 1:600 dilution). MACH4 Universal HRP polymer detection kit mouse/rabbit (M4U534H) from Biocare Medical (Pacheco, CA) was used according to manufacture instructions for the detection of HMGB1 and thioredoxin. DAKO New Fuchsin Substrate System (K698, Dako), was used for the detection of CXCL12 in double-immunohistochemical analysis. Images were acquired using a 40x/0.95 N.A. Plan Apo objective on a Nikon Eclipse E800 upright microscope, equipped with the digital camera DXM1200 (Nikon, Egg, Switzerland), and using Act-1 software version 2.7 (Nikon).

### Extracellular flux assays

All extracellular flux assays were performed using a Seahorse Bioscience XF96 Analyzers. XF glycolysis stress test kit (103020-100), and XF cell mito stress test kit (103015-100), both from Seahorse Bioscience (Agilent, Santa Clara, CA), were used according to the manufacturer instruction. Briefly, monocytes from HD were cultured in Cell Tack-coated plates in the absence or presence of 1 nM PGE_2_ for 6 h. Cells were washed and medium was replaced with the indicated medium for each specific test. Drugs were added at the following final concentrations: glucose (10 mM), 2-D-glucose (50 mM), oligomycin (1 μM), FCCP (0.5 μM), Rotenone/Antimycin A (0.5 μM). ECAR and OCR values were normalized to the reading just before the first drug injection.

### Statistical analysis

Correlation analysis between two groups of values was performed using the Spearman's correlation rank test, determining *r*- and *p*-value. The statistical significance between two groups was determined using the non-parametric Mann-Whitney test. The statistical significance between more than two groups was calculated by using one-way or two-way ANOVA followed by Bonferroni's adjustment.

## Results

### Monocytes from RA patients with active disease require a low concentration of HMGB1 to enhance CXCL12-induced migration

**Figure 1:**
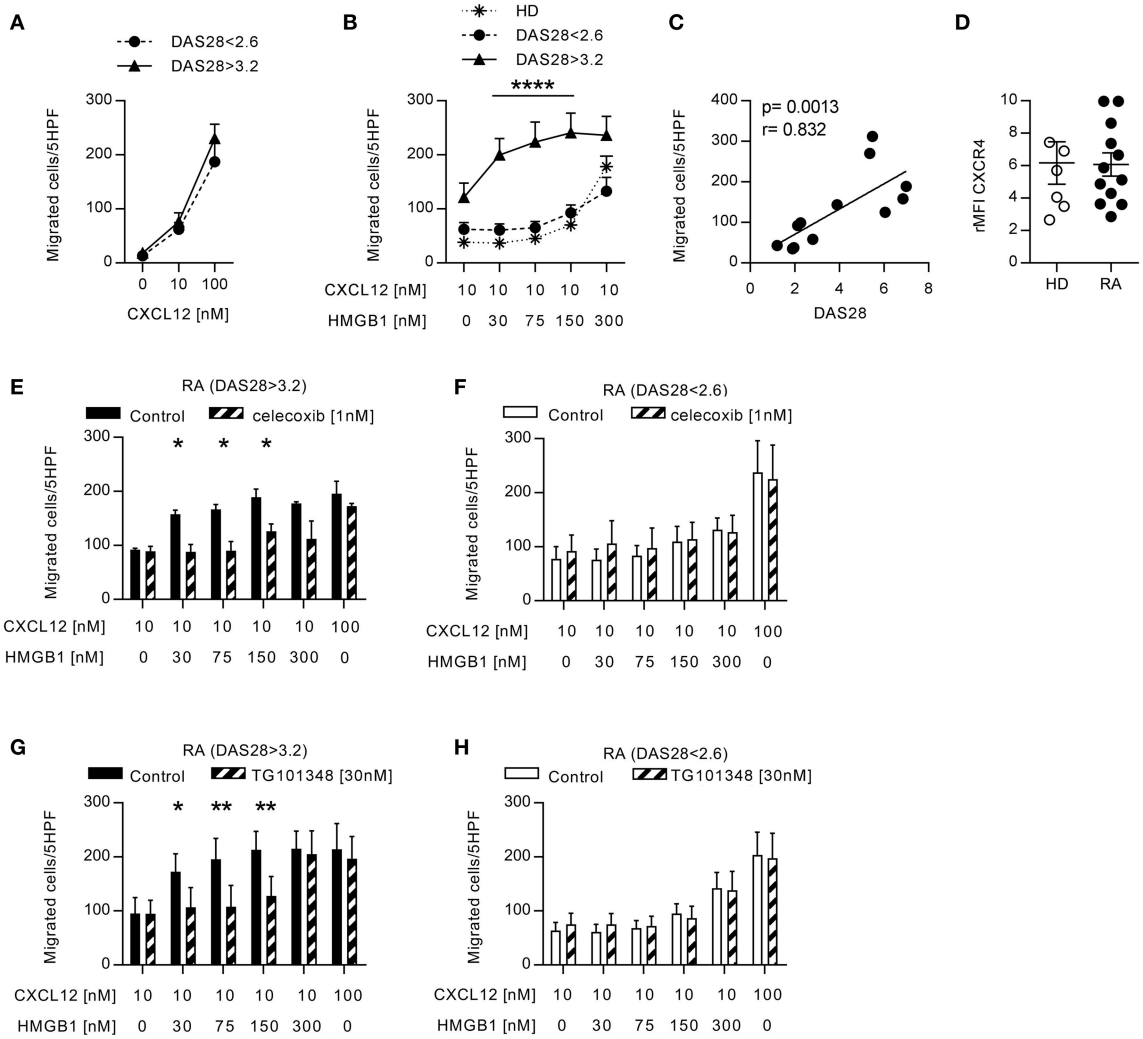
Migration induced by CXCL12 and the CXCL12/HMGB1 heterocomplex in monocytes from patients with RA. **(A)** Migration of monocytes from patients with RA in response to CXCL12 alone. **(B)** Migration of monocytes from 6 RA patients with DAS28>3.2, 6 RA patients in clinical remission, and 5 HD in response to a suboptimal dose of CXCL12 in the absence or presence of increasing concentrations of HMGB1. **(C)** Linear regression analysis performed between the DAS28 index and the number of monocytes from RA patients migrated in response to the heterocomplex formed with 30 nM HMGB1. **(D)** Relative mean fluorescence intensity of CXCR4 expression on freshly isolated monocytes from HD or form patients with RA. **(E-F)** Migration of monocytes from RA patients with DAS28>3.2 **(E)** or DAS28<2.6 **(F)** in response the heterocomplex formed by a suboptimal dose of CXCL12 and increasing concentrations of HMGB1, in the presence or absence of the COX2 inhibitor celecoxib. **(G,H)** Migration of monocytes from RA patients with DAS28>3.2 **(G)** or DAS28<2.6 **(H)** in response to the heterocomplex formed by a suboptimal dose of CXCL12 and increasing concentrations of HMGB1, in the presence or absence of the JAK2 inhibitor (TG101348). In panels **(E–H)** control migrations were performed with a suboptimal or an optimal concentration of CXCL12 alone (10 and 100 nM, respectively). All data are presented as mean±SEM of migrated cells in 5HPF in at least 3 independent experiments performed with cells from different donors. ^*^p < 0.05, ^**^p < 0.01, ^****^p < 0.0001 using two-way ANOVA plus Bonferroni's adjustment or Mann-Whitney test.

The presence of CXCL12 and HMGB1 at high concentrations in the joints and the peripheral blood of patients with RA, could favor the formation of the CXCL12/HMGB1 heterocomplex. Since we previously described that the heterocomplex enhanced monocyte migration on human monocytes from healthy donors (HD) via CXCR4 (21), we evaluated its activity on monocytes from patients with RA, which are exposed to pro-inflammatory stimuli also in the circulation. Migration of freshly isolated monocytes in response to different CXCL12 concentrations, or to a suboptimal concentration of CXCL12 (10 nM) in combination with different concentrations of HMGB1, was assessed *in vitro*. Monocytes from patients with active RA, or in clinical remission, did not differ in their capability to respond to CXCL12 (Figure 1A), and as demonstrated in monocytes from HD (21), they did not migrate in response to HMGB1 alone (data not shown). However, only in patients with active RA a 10-fold lower amount of HMGB1 was required to induce an efficient migration in response to the suboptimal concentration of CXCL12 (Figure 1B). Moreover, we found a significant positive correlation (*r* = 0.832, *p* = 0.0013) between the DAS28 score and the capability of monocytes to migrate in response to the heterocomplex formed with a low concentration of HMGB1 (Figure 1C). This response was not influenced by changes in the expression of CXCR4 on cell surface, as flow cytometric analysis showed no significant differences in CXCR4 expression between monocytes from patients, regardless disease activity, and HD (Figure 1D).

### Identification of the pathways involved in the response to the CXCL12/HMGB1 heterocomplex in RA

To evaluate whether, in RA patients with active disease, the monocyte response to the heterocomplex depends on the activity of pathways that are also target of therapy, we analyzed their migration in the presence of celecoxib, the COX2 inhibitor. The treatment totally abolished the enhanced capability of monocytes from patients with active disease to respond to the heterocomplex (Figure 1E), while it did not affect the migration profile of monocytes from patients in clinical remission (Figure 1F). Notably, both in patients with active disease or in clinical remission, celecoxib did not influence the migration toward CXCL12 alone.

As the JAK-blocking agents represent a novel therapeutic strategy for treating RA patients not responsive to conventional DMARDs, we investigated whether the inhibition of the JAK/STAT pathway could prevent the response observed to the heterocomplex. Monocytes from RA patients with active disease lost their enhanced response to the heterocomplex when cells were treated with a JAK2 selective inhibitor, TG101348, while the migratory response to CXCL12 alone was not affected (Figure 1G), nor the migration profile of monocytes from RA patients in clinical remission (Figure 1H).

These data collectively indicate that the activity of the COX2 and of the JAK2-STAT pathways is necessary to induce the enhanced migratory response to the heterocomplex we observed in monocytes from patients with active RA.

### The response to the heterocomplex in RA is determined by the redox potential in the microenvironment and depends on disease activity

**Figure 2:**
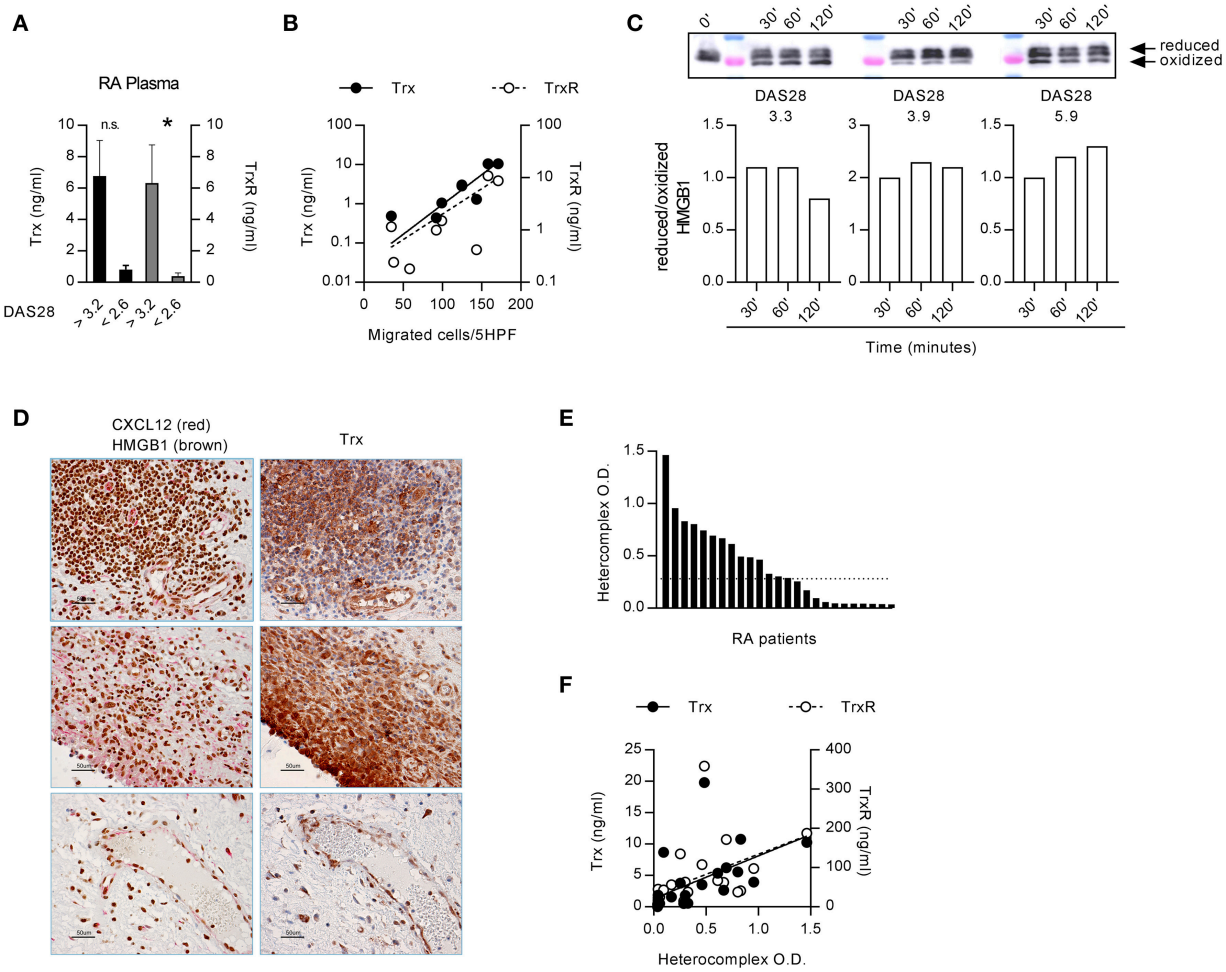
Extracellular redox changes in RA prevent HMGB1 oxidation and promote the heterocomplex formation. **(A)** Plasma levels of Trx and TrxR in RA patients with DAS28>3.2 (*n* = 4) or in clinical remission, DAS<2.6 (*n* = 5). Statistical analysis performed using the Mann-Whitney test (^*^p < 0.05). **(B)** Regression analysis performed between the levels of Trx or TrxR and the number of monocytes from RA patients migrated in response to the heterocomplex formed with 10 nM CXCL12 and 30 nM HMGB1 (Trx: *p* = 0.0038 and *r* = 0.874; TrxR: *p* = 0.058 and *r* = 0.666). **(C)** Analysis of the redox status of HMGB1, at different time points after exposure to the supernatant of monocytes from RA patients with distinct DAS28 scores, performed by Western blot. Reduced over oxidized form of HMGB1 was calculated on the densitometric values of the bands obtained from the experiments performed with supernatants of monocytes from 3 RA patients. **(D)** Representative images of the immunohistochemical analysis performed on the synovial membrane of RA patients with active disease, in three different fields. Panels on the left: expression of CXCL12 (red) and HMGB1 (brown); panels on the right: expression of Trx (brown). All images at 40x magnification (scale bar: 50 μm). **(E)** Presence of the heterocomplex in the synovial fluids of RA patients with active disease, assessed by hybrid ELISA. The dotted line represents the median value. **(F)** Linear regression analysis performed between the levels of Trx or TrxR present in the synovial fluid and the amount of the heterocomplex detected by ELISA (*p* < 0.0001 and *r* = 0.784, *p* = 0.001 and *r* = 0.718, respectively).

The functions of HMGB1, released in the extracellular space, are regulated by different redox states, which confer to protein the ability to form a complex with CXCL12 triggering CXCR4, or to trigger RAGE and TLRs (43). In the microenvironment, HMGB1 is progressively oxidized losing its capacity to form a complex with CXCL12. As a lower HMGB1 concentration is needed to obtain a synergistic effect on monocytes from RA patients with active disease, we investigated the factors that could be responsible for the maintenance of HMGB1 in the reduced state. The thioredoxin system has been described to correlate with disease severity in RA, but its role in the pathogenesis of the disease was not clarified (32, 45). High levels of both Trx and TrxR were present in plasma from RA patients with active disease (Figure 2A). Moreover, the plasma levels of TrxR positively correlated with the DAS28 (TrxR: *r* = 0.781, *p* = 0.0172) and Trx correlated with monocytes ability to migrate in response to the heterocomplex formed by CXCL12 with 30 nM HMGB1 (Figure 2B; Trx: *p* = 0.0038 and *r* = 0.874; TrxR: *p* = 0.058 and *r* = 0.666).

Primary monocytes from HD express good amount of Trx that increases upon activation, and higher levels of Trx are present in unstimulated monocytes from patients affected by pathologies such as autoinflammatory diseases (46). We therefore hypothesized that in RA patients with active disease, the thioredoxin system maintains HMGB1 in the reduced form, which binds CXCL12 enhancing its activity. Indeed, depending on the DAS28 score, monocytes from RA patients showed a different degree of oxidative capacity, with the reduced form of HMGB1 preserved for a prolonged period of time, when exposed to the supernatant of monocytes from patients with the higher DAS28 (Figure 2C). These data are in agreement with the correlation we observed between migration induced by the heterocomplex and the DAS28 score (Figure 1C).

Notably, HMGB1, CXCL12, and Trx were all present in the same area of the synovial membranes of RA patients (Figure 2D). In particular, synoviocytes, infiltrating cells, and endothelial cells all expressed high levels of Trx. The heterocomplex was also detected, at different levels, in the synovial fluid of patients with active disease (Figure 2E), and it positively correlated with local Trx and TrxR concentrations (Figure 2F; Trx: *p* < 0.0001 and *r* = 0.784; TrxR: *p* = 0.001 and *r* = 0.718, respectively).

### PGE_2_ stimulation of monocytes from HD recapitulates the response of monocytes from patients with active RA

**Figure 3:**
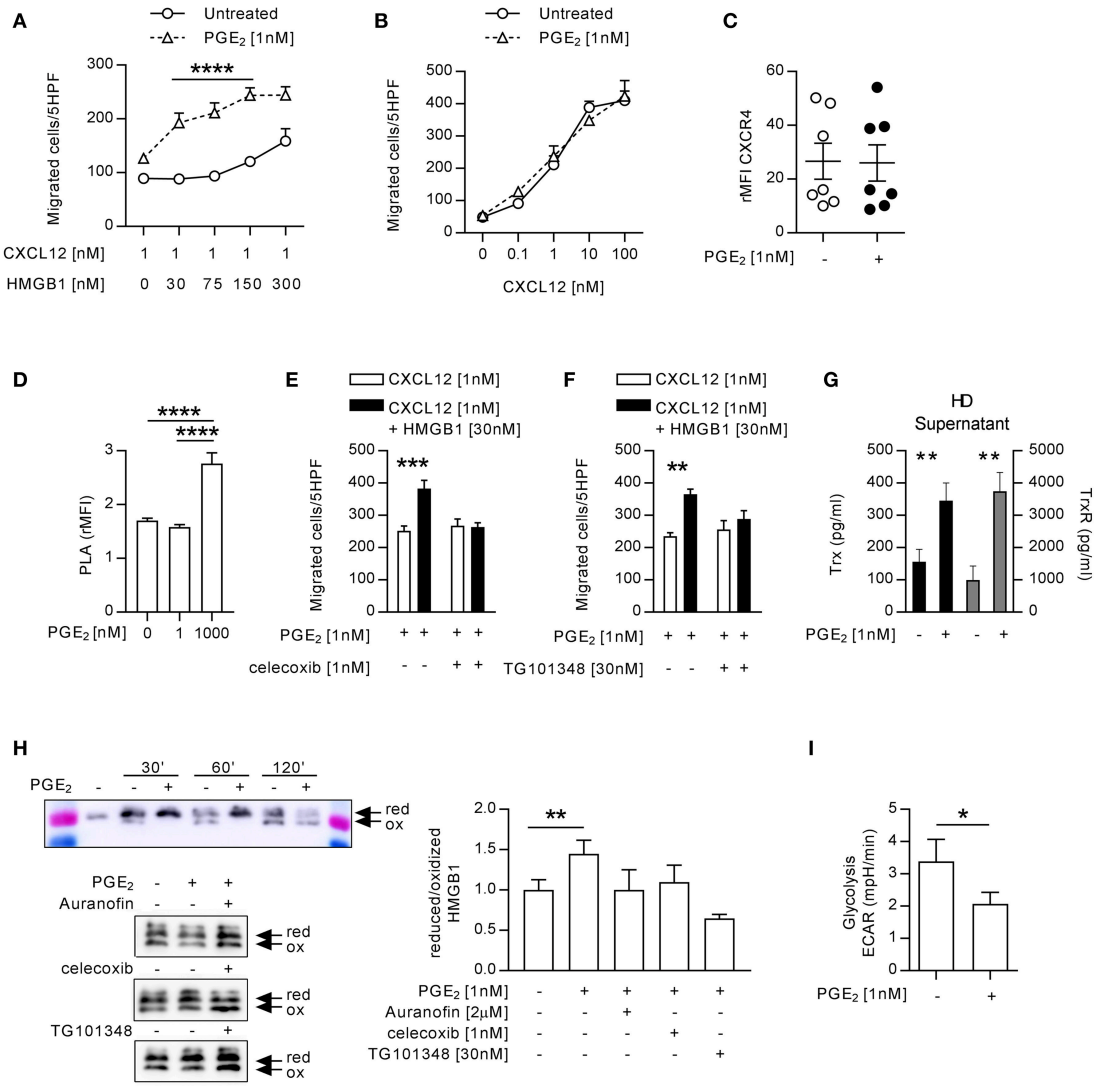
Monocytes from HD treated with 1 nM PGE_2_ recapitulate the response of monocytes from RA patients with active disease. **(A)** Migration of monocytes (*n* = 5) untreated or treated with PGE_2_ in response to a suboptimal dose of CXCL12 in the absence or presence of increasing concentrations of HMGB1. Data are presented as mean±SEM of migrated cells in 5HPF. **(B)** Migration of HD monocytes untreated or treated with PGE_2_ in response to CXCL12 alone. **(C)** Relative mean fluorescence intensity of CXCR4 expression on HD monocytes untreated or treated with PGE_2_. Statistical analysis was performed using the Mann-Whitney test. **(D)** Relative mean fluorescence intensity of CXCR4 oligomerization, determined by PLA, in HD monocytes untreated or treated with 1 or 1,000 nM PGE_2_. Statistical analysis was performed using the one-way ANOVA plus Bonferroni's adjustment: ^****^p<0.0001. **(E)** Migration of monocytes from HD treated with PGE_2_ in response to CXCL12 or the heterocomplex, in the presence (*n* = 4) or absence (*n* = 6) of the COX2 inhibitor celecoxib. **(F)** Migration of monocytes from HD treated with PGE_2_ in response to CXCL12 or the heterocomplex, in the presence (*n* = 3) or absence (*n* = 3) of the JAK2 inhibitor (TG101348). Data in panels **(A,B,E,F)** are presented as mean±SEM of migrated cells in 5HPF, in at least three independent experiments, and were analyzed using two-way ANOVA plus Bonferroni's adjustment: ^**^p < 0.01, ^***^p < 0.001, ^****^p < 0.0001. **(G)** Levels of Trx and TrxR in supernatant of monocytes (*n* = 11) untreated or treated with PGE_2_. Statistical analysis was performed with the Mann-Whitney test (^**^p < 0.01). **(H)** Analysis of the redox status of HMGB1, at different time points after exposure to the supernatant of monocytes from HD, untreated or treated with PGE_2_, and in the presence of Auranofin, celecoxib or TG101348 was performed by Western Blot. Representative images on the left panels. Reduced over oxidized form of HMGB1 was calculated on the densitometric values of the bands obtained from the experiments performed with supernatants of monocytes from 3 HD. Data are presented as mean±SEM, and were analyzed using the Mann-Whitney test (^**^p < 0.01). **(I)** Glycolytic metabolism of monocytes from 6 HD untreated or treated with PGE_2_, expressed as extracellular acidification rate (ECAR) in mpH/min. Statistical analysis was performed with the Mann-Whitney test (^*^p < 0.05).

The exposure of monocytes to pro-inflammatory mediators in the bloodstream is a common characteristic of patients with autoimmune diseases, and could be responsible for the activity of the heterocomplex we have observed in monocytes from patients with active RA. Therefore, we treated monocytes from HD with different pro-inflammatory stimuli (IL-1β, IL-6, or PGE_2_) and tested their ability to migrate in response to CXCL12 or to the heterocomplex. While we did observe a significant reduction in cell migration in response to CXCL12 and to the heterocomplex when monocytes were treated with IL-1β or IL-6 (Supplementary Figure 1), monocytes exposed to 1 nM PGE_2_ recapitulated the responses observed in monocytes from RA patients with active disease (Figures 3A,B and Supplementary Figure 1D). Of note, in monocytes freshly isolated from blood, part of the CXCR4 is internalized and is re-expressed on the cell surface upon culture. The higher surface expression of CXCR4 in cultured monocytes compared to freshly isolated cells shifts their responsiveness to lower CXCL12 concentrations, thus changing accordingly the suboptimal concentration of the chemokine. The enhanced response to the heterocomplex, we observed in monocytes cultured in the presence of PGE_2_, was not due to changes in the expression of CXCR4, as assessed by flow cytometry (Figure 3C), nor in the frequency of CXCR4 homodimers on the cell surface, known to increase at high PGE_2_ concentrations, quantified by confocal microscopy using the proximity ligation assay (Figure 3D). The PGE_2_ treatment did not alter the expression of TLR2 and TLR4, while RAGE was never detected on the surface of untreated or treated monocytes (data not shown).

The stimulation with 1 nM PGE_2_ induced also the release of picogram amounts of PGE_2_ (207 ± 86 pg/ml; mean±SEM) in a feed-forward loop, in line with the observation that the COX2 inhibitor affects monocytes responses in patients with active disease. Indeed, celecoxib abolished in a similar manner the response of monocytes from HD pre-treated with PGE_2_, without affecting the response to CXCL12 alone (Figure 3E). Moreover, also the JAK2 selective inhibitor abolished the response of monocytes from HD pre-treated with PGE_2_ (Figure 3F). As shown in Figure 3G, supernatants of monocytes from HD pre-treated with PGE_2_ contained significantly higher levels of Trx and TrxR, than untreated monocytes and, similarly to what observed in RA patients, HMGB1 was preserved in the reduced form when exposed to supernatant from PGE_2_ treated monocytes (Figure 3H). To test the role of the thioredoxin system in the regulation of the HMGB1 redox status in the extracellular space, we specifically inhibited the TrxR activity in monocytes from HD pre-treated with PGE_2._ Indeed, the TrxR inhibitor Auranofin led to a fast oxidation of HMGB1, similarly to what observed in the supernatant of untreated monocytes, proving the relevance of the thioredoxin system in the modulation of the redox status of HMGB1. Both celecoxib and the JAK2 selective inhibitor, TG101348 promoted HMGB1 oxidation in the supernatant of monocytes pre-treated with PGE_2_ (Figure 3H).

Monocytes exposed to PGE_2_ showed a reduced glycolysis, whereas their oxygen consumption was not altered (Figure 3I and Supplementary Figure 2), suggesting also a metabolic reprogramming aimed at counteracting the acidification of the extracellular space induced by the inflammatory stimulus.

## Discussion

This study has focused on the interaction of CXCL12 and HMGB1 in RA, in which the individual contribution of different pro-inflammatory mediators is yet not fully understood. The response of peripheral monocytes to the heterocomplex formed with low concentration of HMGB1 can be maintained in RA by the activity of essential players: the PGE_2_/COX2 pathway, the JAK/STAT pathway, and the thioredoxin system, all associated to active disease.

The relevance of molecules that can synergize with chemokines has never been evaluated on human leukocytes from patients with inflammatory disorders. The first indications of synergy between chemokines came with the discovery of CXCL4 as amplifier of CCL5 activities (47), of several chemokines as enhancers of CCL19- and CCL21-induced responses (48), and of CXCL10 as amplifier of CCL22 activities (49). Some years later, a model of human atherosclerosis showed how crucial is disrupting the formation of a chemokine-heterocomplex. A small peptide disrupting the CXCL4/CCL5 heterocomplex was able, alone, to reduce the formation of atheromatic plaques (50). We demonstrated a functional synergism between CXCL12 and the alarmin HMGB1 in a mouse model of sterile tissue damage. Inhibition of the formation of the heterocomplex, obtained with glycyrrhizin, resulted in a significant reduction of monocytes influx into the muscle (21). So far, no data were available on the presence of heterocomplexes in human pathology, nor their activity was tested on leukocytes from patients.

The present work shows that monocytes from patients with active RA respond to the heterocomplex CXCL12/HMGB1 formed in the presence of low HMGB1 concentrations. In HD and RA patients in clinical remission, the presence of low HMGB1 concentrations is not sufficient to enhance CXCL12-induced responses. These novel data indicate that the synergism between these molecules could influence the progression of the disease by enhancing the migration of monocytes to site of inflammation. The association between PGE_2_ and RA inflammatory processes is well-known, also due to the ability of PGE_2_ to promote the expression of pro-inflammatory cytokines (51). In fact, many inhibitors of PGE_2_ biosynthesis have been developed and show good pharmacological efficacy (29). Here we demonstrate that the COX2 inhibitor, celecoxib, can convert the response to the heterocomplex of monocytes from patients with active disease, to the response observed in patients in clinical remission or in HD.

The activation of JAK-STAT signaling cascade is important for the function of CXCR4 (52), the selective receptor used by the heterocomplex. Inhibitors of the JAK-STAT signaling cascade have also been used in the treatment of RA because of the crucial role of JAK in the signaling of the type I and type II inflammatory cytokine receptors (28, 53). JAK2 phosphorylation has been shown to be higher in monocytes from RA patients with active disease as compared to patients in remission (54). JAK2 activation in monocytes from RA patients with active disease is also required for achieving the response to the heterocomplex. Nonetheless, JAK2 inhibition does not modify the activity of the heterocomplex when high concentrations of HMGB1 are applied, suggesting that in this condition, CXCR4 triggering can lead to the activation of additional pathways. The concomitant activation of the COX2/PGE_2_ and JAK/STAT pathways is able to promote the response to the heterocomplex formed with low concentration of HMGB1, and provides evidence of the intricate interplay between these two signaling cascades in promoting monocyte migration in patients with active RA.

**Figure 4:**
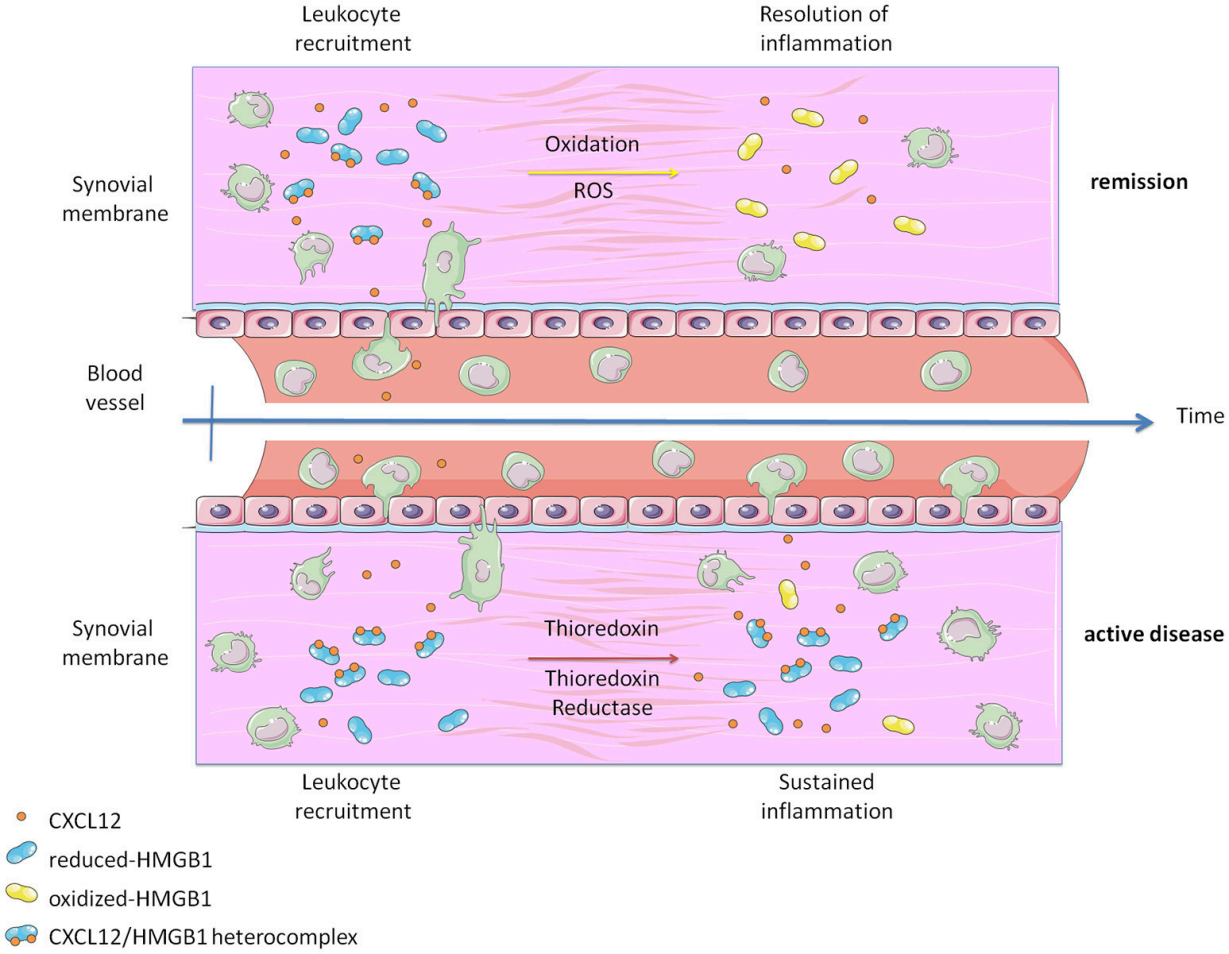
Graphical abstract representing how the heterocomplex CXCL12/HMGB1 is maintained in active Rheumatoid Arthritis thanks to the activity of the Thioredoxin system. The panel was created using Servier Medical Art according to Creative Commons Attribution 3.0 Unported License (https://creativecommons.org/licenses/by/3.0/). Changes were made to the original cartoons.

Inflammatory conditions are associated with the release of Reactive Oxygen Species (ROS), which alters the redox status of the microenvironment. Our data suggest that the oxidative stress induced by the exposure of monocytes to PGE_2_ is balanced by the release of Trx and TrxR, and by a reduction in the glycolytic metabolism in order to counteract the acidification of the extracellular space. In active RA, we have observed the release of high levels of Trx and TrxR, produced by blood vessels and synoviocytes, which represents an attempt of reducing the oxidative stress. Nonetheless, this pathway becomes detrimental when HMGB1 is released, because the protein is preserved in its reduced form, contributing to fuel the income of inflammatory monocytes into the synovium (Figure 4). Of note, reduced HMGB1 is not sufficient to trigger migration via CXCR4, but needs the concomitant presence of CXCL12. Additional proteins associated with the membrane and important for cell adhesion, such as the integrin α4, could be also affected by changes in the redox status of the microenvironment and further contribute to the enhanced migration (55).

The histologic features of the synovial membrane in patients with RA can vary from follicle-like-structures rich phenotype to a more diffuse inflammatory infiltrate with prevalence of macrophages. We have found the heterocomplex at different levels in the synovial fluids of patients with active disease. Additional studies aiming at evaluating the correlation between the histologic pattern of the RA synovitis and the levels of the CXCL12/HMGB1 heterocomplex in the joint fluids are needed, and might reveal a preferential expression of the heterocomplex in macrophage-rich infiltrates. If our hypothesis would be confirmed, the heterocomplex might be used as a biomarker of synovial inflammatory infiltrate arrangement, guiding novel personalized therapeutic approaches.

Thus, the chemokine system remains a promising biological target for the development of new therapeutic tools for the treatment of chronic diseases, even if some limitations hamper the efficacy of conventional competitive inhibitors. The formation of heterocomplexes that enhance chemokine activities calls for innovative approaches (56, 57). Novel data indicate that CXCL12/HMGB1 promotes tissue regeneration (23, 24), and together with the present findings demonstrate how diverse can be the activity of the heterocomplex if associated to tissue damage or to a state of cell activation in the presence of a pathological condition. Tissue regeneration and tissue damage could be also part of the same disease, like suggested for Ankylosing Spondylitis (AS), where spinal bone formation occurs as a post-inflammation tissue remodeling reaction rather than an uncoupled process from inflammation (58). Future studies will clarify if post-inflammation tissue remodeling reactions could be ascribed to the activity of this heterocomplex. The specific conformational changes induced in CXCR4 by the heterocomplex (21), the prevention of CXCL12 degradation when the chemokine is complexed with HMGB1 (59), the various signaling cascades triggered by the heterocomplex in active RA, all need consideration when novel therapeutic strategies for chronic inflammatory diseases will be exploited.

## Author contributions

VC, GiD, LR, and MS designed and performed most of the experiments. GaD participated in some of the experiments. VC, AN, AM, MT, MB, AC, AR, CP, and MU discussed results and participated in the writing of the manuscript. VC, GiD, and MU wrote most of the manuscript, and MU was responsible for general organization.

### Conflict of interest statement

MB is founder and part owner of HMGBiotech, a company that provides goods and services related to HMGB proteins. The remaining authors declare that the research was conducted in the absence of any commercial or financial relationships that could be construed as a potential conflict of interest.
