# Supplementary material for: Redox-Mediated Mechanisms Fuel Monocyte Responses to CXCL12/HMGB1 in Active Rheumatoid Arthritis
Source: Front Immunol. 2018 Sep 19;9:2118. doi: 10.3389/fimmu.2018.02118 (PMC6157448; doi:10.3389/fimmu.2018.02118)
Supplement: Supplementary file 1 [file Data_Sheet_1.docx]

Supplementary Material

Redox-mediated mechanisms fuel monocyte responses to CXCL12/HMGB1 in active Rheumatoid Arthritis

Valentina Cecchinato, Gianluca D’Agostino, Lorenzo Raeli, Alessandra Nerviani, Milena Schiraldi, Gabriela Danelon, Antonio Manzo, Marcus Thelen, Adrian Ciurea, Marco E. Bianchi, Anna Rubartelli, Costantino Pitzalis, and Mariagrazia Uguccioni^*^

*** Correspondence:** Dr. Mariagrazia Uguccioni, Institute for Research in Biomedicine, Faculty of Biomedical Sciences, Università della Svizzera italiana, Bellinzona, Switzerland
[mariagarzia.uguccioni@irb.usi.ch](mailto:mariagarzia.uguccioni@irb.usi.ch)

##
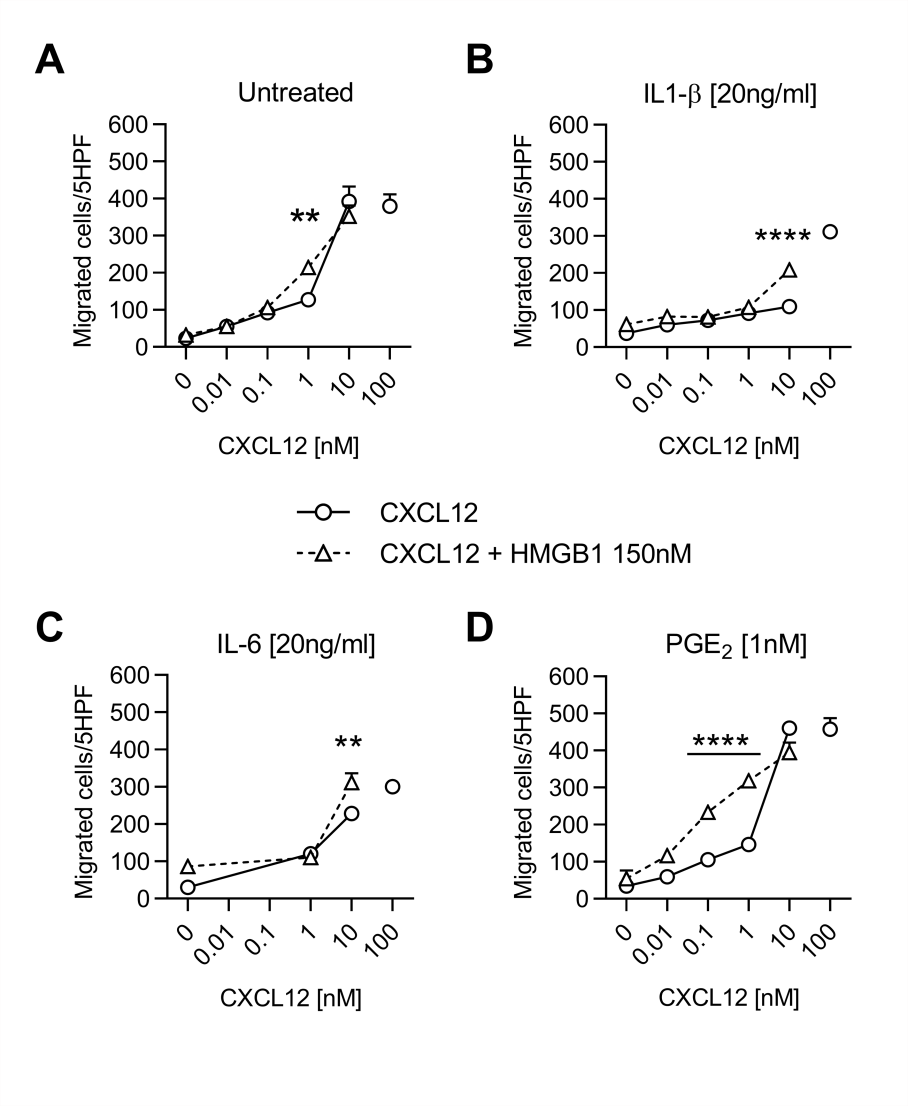


**Supplementary Figure 1.** Migration induced by the heterocomplex in monocytes from HD upon pro‑inflammatory treatments. Migration of HD monocytes untreated **(A)**, or treated for 6 h with IL‑1β **(B)**, IL‑6 **(C)** or PGE_2_ **(D)**, in response to increasing concentrations of CXCL12 in the absence or presence of 150 nM HMGB1. All data are presented as mean±SEM of migrated cells in 5HPF in at least 3 independent experiments performed with cells from different donors.

**p<0.01, ****p<0.0001 using two-way ANOVA plus Bonferroni’s adjustment.


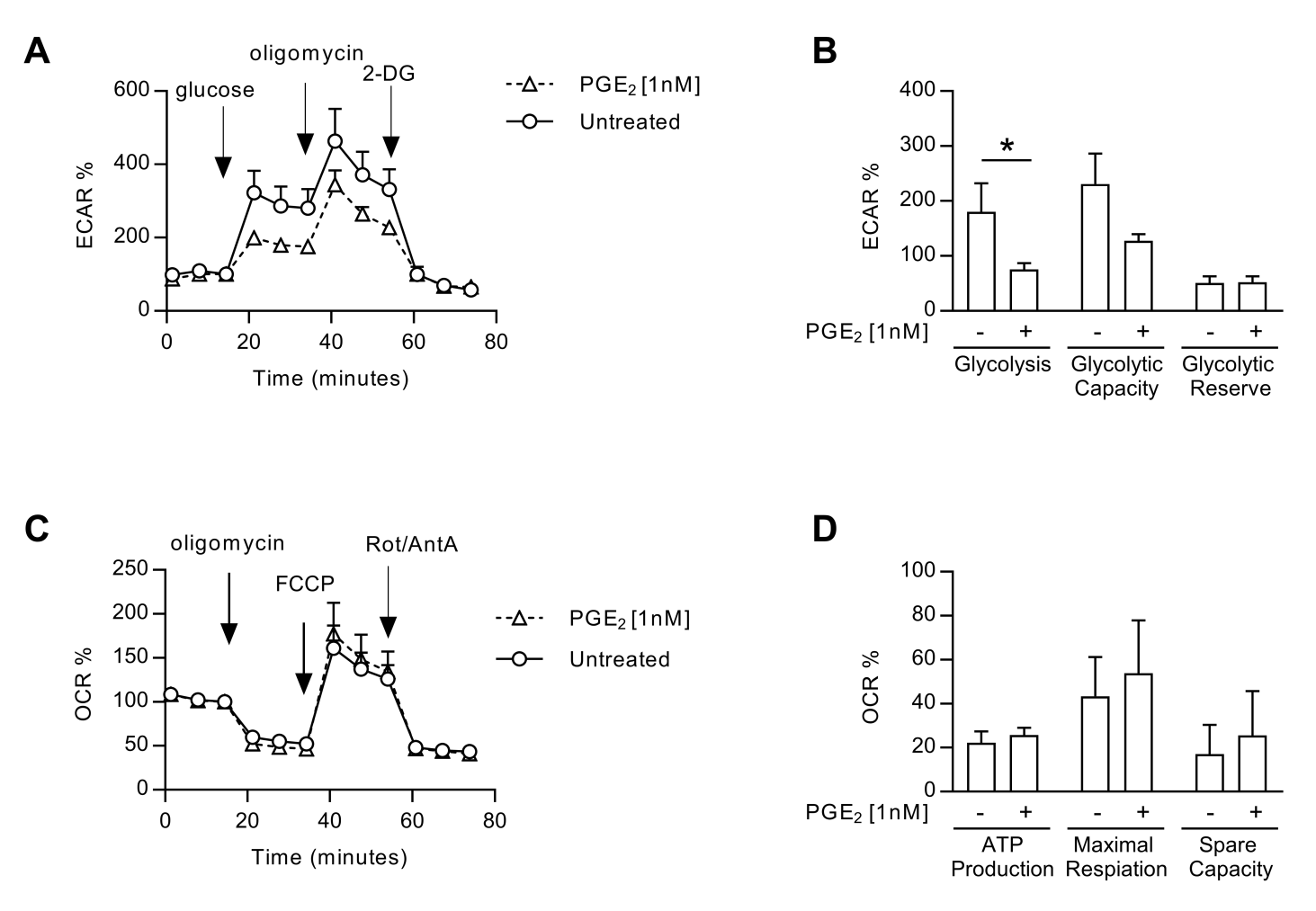


**Supplementary Figure 2.** Monocytes from HD treated with 1 nM PGE_2_ reduce their glycolytic metabolism, without changing the oxygen consumption rate (OCR). Extracellular flux analyses in monocytes from HD untreated or treated with PGE_2_ were analyzed. **(A)** Extracellular acidification rate (ECAR) was measured in real-time under basal conditions and in response to the injection of the indicated drugs. **(B)** Glycolysis, glycolytic capacity and glycolytic reserve were calculated from the ECAR values. **(C)** Oxygen consumption rates (OCR) were measured in real-time under basal conditions and in response to indicated mitochondrial inhibitors. **(D)** ATP production, maximal respiration and spare capacity were calculated from the OCR values. ECAR and OCR values are normalized to the reading just before the first drug injection. Data are presented as mean±SEM of 6 independent experiments. Statistical analysis was performed with the Mann-Whitney test (*p<0.05).
